# The central-peripheral dichotomy and metacontrast masking

**DOI:** 10.1177/03010066221108281

**Published:** 2022-07-18

**Authors:** Li Zhaoping, Yushi Liu

**Affiliations:** 234487 University of Tübingen, Tübingen, Germany; 28328 Max Planck Institute for Biological Cybernetics, Tübinge, Germany; 234487 University of Tübingen, Tübingen, Germany

**Keywords:** Attention, crowding/eccentricity, neural mechanisms, object recognition

## Abstract

According to the central-peripheral dichotomy (CPD), feedback from higher to lower cortical areas along the visual pathway to aid recognition is weaker in the more peripheral visual field. Metacontrast masking is predominantly a reduced visibility of a brief target by a brief and spatially adjacent mask when the mask succeeds rather than precedes or coincides with the target. If this masking works mainly by interfering with the feedback mechanisms for target recognition, then, by the CPD, this masking should be weaker at more peripheral visual locations. We extended the metacontrast masking at fovea by Enns and Di Lollo to visual field eccentricities 1 ${}^{\circ }$ , 3 ${}^{\circ }$ , and 9 ${}^{\circ }$ . Relative to the target’s onset, the mask appeared at a stimulus onset asynchrony (SOA) of −50 , 0, 50, 92, or 142 milliseconds (ms). Enlarged stimuli were used for larger eccentricities to equalize target discrimination performance across eccentricities as best as possible for zero SOA and when SOA was too long for substantial masking. At each eccentricity, the masking was weakest at 0 or −50 ms SOA, strongest at 50 ms SOA, and weakened with larger (positive) SOAs. Consistent with the CPD, larger eccentricities presented weaker maskings at all nonzero, and particularly the positive, SOAs.

## Introduction

The central-peripheral dichotomy (CPD) (Zhaoping, 2017, 2019) is a recent proposal motivated mainly by the following two observations. One is the presence of an attentional bottleneck for visual recognition and the other is an increasing level of experimental support to the V1 saliency hypothesis (V1SH) that a saliency map is created in the primary visual cortex (V1) to guide attention or gaze shifts exogenously (Li, 2002), see review in Zhaoping (2014). The attentional bottleneck means that, due to limited brain resources, only a tiny fraction of all visual input information is selected for deep processing or visual recognition. This selection is often via gaze shifts to the selected location or object, and V1SH implies that the selection should start by V1’s output (Zhaoping, 2019). Accordingly, visual information from V1 to higher visual areas along the visual pathway is impoverished, giving ambiguous information about visual objects to be recognized. To aid object recognition in ambiguous or challenging situations (such as brief viewing durations, noisy inputs, or partially occluded objects), feedback from higher to lower visual cortical areas could query for additional information using analysis by synthesis as part of a perceptual decision-making process. This feedback query works as follows: first, the higher visual areas synthesize the would-be sensory signals according to the initial perceptual hypotheses about the sensory scene; then, the synthesized signals are fed back and compared with the ongoing sensory signals in early visual areas to update the hypotheses to arrive at an ultimate perceptual outcome. The CPD states that this feedback is mainly directed to the central fovea, which is typically centered on the object selected by attention to be recognized (Zhaoping, 2017, 2019). Hence, peripheral vision relies mainly or only on feedforward visual inputs for recognition, making it more vulnerable than central vision to visual illusions that could arise from impoverished and misleading visual inputs. The CPD is consistent with the observations that many visual illusions, including the rotating snake illusion (Hisakata and Murakami, 2008), the Hermann grid illusion (Schiller and Carvey, 2005), the furrow illusion (Anstis, 2012), the curved ball illusion (Shapiro et al., 2010), and the reversed Phi motion illusion (Anstis, 1970), tend to be stronger or only occur in the peripheral visual field. Knowledge of V1’s neural properties has also enabled the CPD to predict two new illusions, reversed-depth in contrast-reversed random-dot stereograms (Zhaoping and Ackermann, 2018) and filt tilt illusions (Zhaoping, 2020), that are subsequently confirmed experimentally to typically occur only in the peripheral but not central visual field.

The CPD also suggests that, if an illusion or phenomenon is associated with top-down feedback for recognition, then it should be stronger foveally (Zhaoping, 2019). This paper applies this prediction to metacontrast masking. Metacontrast masking is predominantly a reduction in the visibility of a brief target by a brief and spatially adjacent mask when the mask succeeds rather than precedes or coincides with the target, and the strongest masking occurs when the mask appears around 40–100 ms after the target appears (Kahneman, 1968; Enns and Di Lollo, 1997; Breitmeyer and Öğmen, 2006). It has been controversial whether predominantly feedforward or feedback mechanisms for visual recognition are interfered with by metacontrast masking. The masking effect is dramatically weakened by slightly increasing the distance between the target’s contour and the mask’s contour, supporting the idea that the masking works by inhibition of the neurons responding to the target by nearby neurons responding to the mask along the feedforward route (Breitmeyer and Öğmen, 2006; Macknik and Martinez-Conde, 2007). However, neurophysiological recording from monkey V1 (Bridgeman, 1980) and also from V2 (von der Heydt, 2022) and visual evoked potentials at scalp (Jeffreys and Musselwhite, 1986) showed that, very soon after the target’s onset, early cortical responses to masked and unmasked targets are similar. These observations suggest that masking had a limited effect on early visual cortical responses, consistent with the idea that masking interfered with the feedback processes to perceive the target.

In comparison to metacontrast masking, pattern masking and object substitution masking (OSM) are less controversially believed to interrupt, respectively, feedforward and feedback mechanisms for target recognition (Enns and Di Lollo, 2000). Pattern masking occurs when the target contours and mask contours overlap spatially, whereas OSM is often examplified by the four-dot masking (Enns and Di Lollo, 2000) in which the mask comprises four dots surrounding but sufficiently away from the target. OSM is typically observed by a common onset for the target and the mask, and the masking effect is typically weak unless the mask’s offset is delayed after the target’s offset and when an observer’s attention is not properly focused on the target at the beginning of the target’s presentation because the observer is uncertain about the target’s location before its appearance (Enns and Di Lollo, 2000). The mechanisms behind OSM have been proposed as follows (Enns and Di Lollo, 2000; Di Lollo et al., 2000): an initial feedforward processing of visual input along the visual pathway (including V1 and higher brain areas) generates initial perceptual hypotheses about the visual inputs—the target and the mask—in higher brain areas; these hypotheses require comparison with the high-resolution sensory information in V1 via subsequent feedback to V1; when the feedback signals arrive at V1 there is a mismatch between the initial hypotheses and the on-going V1 activities signalling information about the trailing mask alone; this mismatch causes the initial hypotheses to be substituted by new hypotheses about the mask alone, thus generating the masking effect. This OSM proposal is supported by experimental data. Event-related potentials from human scalp suggests that the target triggers a shift of attention to it, however, by the time attention is shifted to the target only the mask remains visible (Woodman and Luck, 2003). Data from functional magnetic resonance imaging (Weidner et al., 2006) indeed show that V1 and some higher brain areas that are plausibly involved in perceptual hypothesis processing have higher neural activities when the masking is effective, presumably to process the mismatch.

There are some similarities between metacontrast masking and OSM (Goodhew et al., 2013). In particular, both types of masking are unlike pattern masking such that the mask and target contours do not spatially overlap each other and that the masking effect is substantial by a trailing rather than a preceding mask. Furthermore, the early visual cortical responses do not seem to distinguish between masked and unmasked situations (Bridgeman, 1980; Jeffreys and Musselwhite, 1986; von der Heydt, 2022; Woodman and Luck, 2003) in both metacontrast masking and OSM. One could then ask whether metacontrast masking also interferes with the feedback mechanisms. However, notable differences between metacontrast masking and OSM are apparent. Metacontrast masking works only when the mask’s contour is very close to the target’s contour, while OSM is insensitive to the distance between these contours (Enns and Di Lollo, 1997). Visual crowding (Levi, 2008; Whitney and Levi, 2011), the deficit in identifying a target in visual periphery when this target is surrounded by flankers, can be reduced when the flankers are masked by metacontrast masking but not by OSM masking, suggesting that metacontrast masking but not OSM may act earlier than crowding (Chakravarthi and Cavanagh, 2009) in the stages of visual processing.

This paper uses the CPD to ask whether metacontrast masking also involves disrupting the feedback mechanisms for target recognition. If the answer is yes, the CPD would predict that this masking should be weaker at more peripheral visual locations. To test this prediction, we adapt the metacontrast masking stimulus from Enns and Di Lollo (1997) for visual locations at three different visual eccentricities, 1 ${}^{\circ }$ , 3 ${}^{\circ }$ , and 9 ${}^{\circ }$ . Enns and Di Lollo showed a strong role of attention in OSM (Di Lollo et al., 2000; Enns and Di Lollo, 1997), such that the masking is stronger (also for metacontrast masking) when observers could not predict the target location before the target appears. To answer our question on whether the feedback mechanisms are involved, we minimize the role of attention by making target’s position (eccentricity) certain for observers before each trial. Visual crowding, which is stronger at visual locations of a larger eccentricity, may also have contributed to impair target recognition in the metacontrast masking and four-dot masking experiments by Enns and Di Lollo, causing poor performance even at zero stimulus onset asynchrony (SOA) between the target and mask (Enns and Di Lollo, 1997). Our study removes this crowding factor by making the stimulus larger when they are presented at a larger eccentricity, so that target discrimination has comparable performance across eccentricities when SOA is too small or too large for masking.

In anticipation, we found that at all three eccentricities, target discrimination performance as a function of SOA followed a U-shaped curve that depressed mainly at positive, small, SOAs, as is characteristic of metacontrast masking for central vision. However, larger eccentricities yielded better task performance and a faster recovery of the target discrimination performance with increasing SOA, as predicted by the CPD if metacontrast masking mainly interrupts the feedback mechanisms.

## Materials and Method

**Figure 1.**
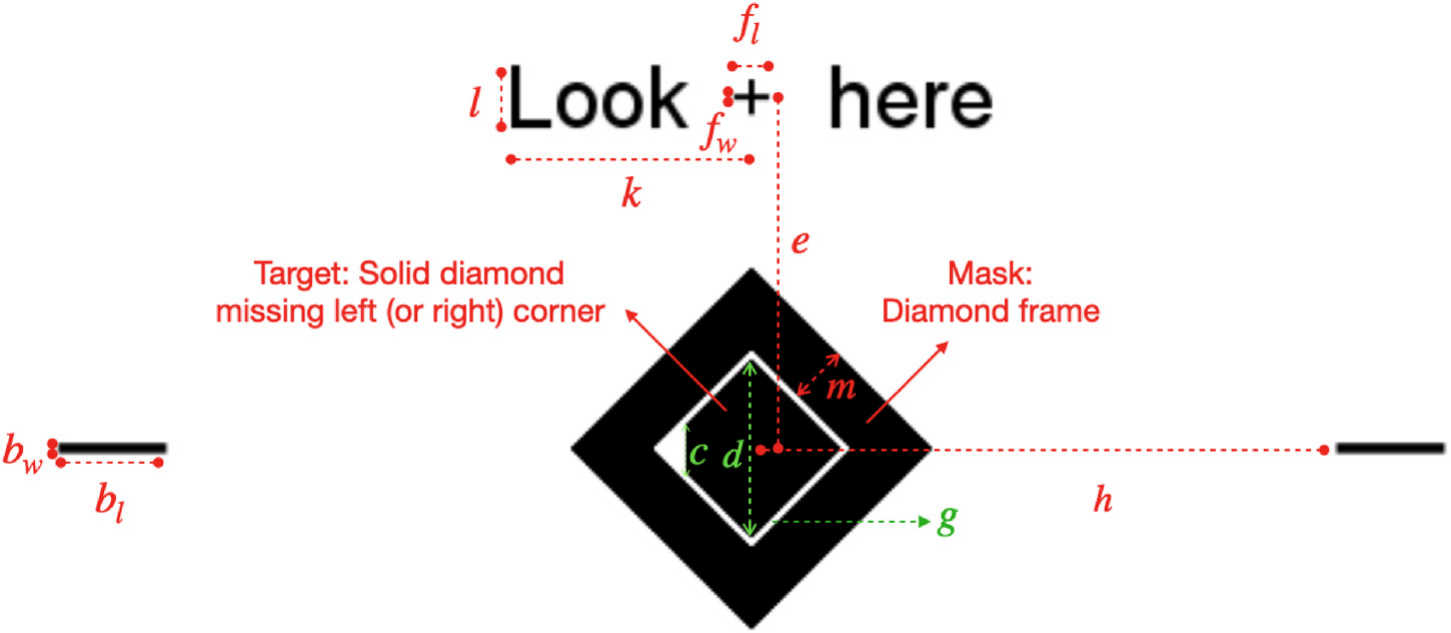
The spatial positioning of the experimental stimuli, and the notations for various sizes and spatial extents. The target diamond missing its left or right corner, the mask surrounding the target, the fixation cross, the horizontal bars, and the text string “Look here” are all black on a white background. All the colored markings and colored texts indicate the positions and sizes of various stimulus components, and are not part of the stimuli. Participants’ task was to report whether the target diamond missed a left or right corner. “Look here” appeared only at the beginning of each trial to prompt subject to fixate on the cross. The fixation cross and the horizontal bars were displayed on the screen throughout each trial to anchor the target diamond’s center location horizontally and vertically. The fixation cross was at the same location on the display throughout an experimental session, while the eccentricity e of the target diamond (and thus the vertical location of the horizontal bars) varied randomly from trial to trial. The sizes and spatial positions are indicated by d (length of the target diamond’s diagonal line), e (eccentricity, center-to-center distance between the target diamond and the fixation cross), c (the vertical extent of the missing corner in the target diamond), g (the width of the white gap between the target and mask), m (the width of the mask diamond’s frame), h (shortest distance between each horizontal bar and the center of the target diamond), $b_{l}$ / $b_{w}$ (length/width of each horizontal bar), $f_{l}$ / $f_{w}$ (length/width of each bar in the fixation cross), l (height of the text font for “Look here”), and k (the rough horizontal extent of “Look” and “here”).

**Figure 2.**
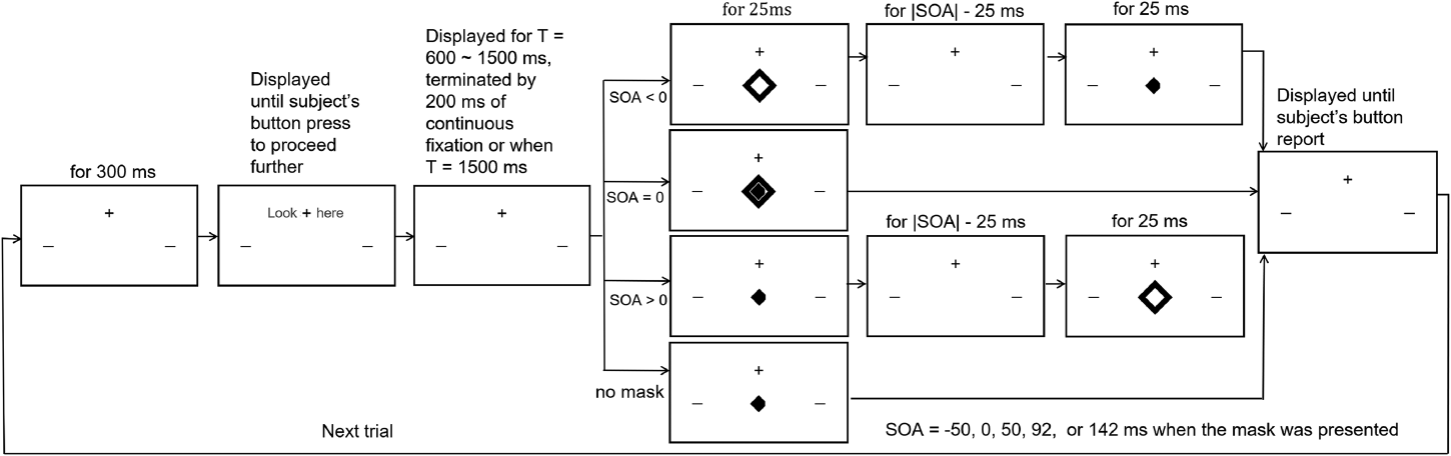
Temporal sequence of events in an experimental trial. For illustrative purposes, the sizes of stimulus elements drawn here are not scaled exactly as in the experiment. There were 18 possible conditions made from combinations of six possible target-mask situations (five stimulus onset asynchrony (SOAs) and one no-mask situation) and three possible eccentricities $e=1^{\circ }$ , $3^{\circ }$ , and $9^{\circ }$ . Each condition had 40 trials. Trials of the 18 conditions were randomly interleaved for a total of eight blocks of 90 trials each.

A total of 30 observers (experimental subjects, 12 male) with normal or corrected vision participated in the experiment whose results are reported in the figures of this paper. All except one of them were naive to the purpose of the experiment. Their minimum, maximum, and average ages were 20, 36, and 26.7 years old, respectively. One of the authors was always present with each participant throughout an experimental session. The target and mask stimuli were adapted from that of Enns and Di Lollo (1997). The target was a black solid diamond (a square with each of its sides tilted $\pm 45^{\circ }$ from horizontal) missing its left or right corner. The mask was a black diamond frame surrounding and not overlapping with the target (see Figure 1). There were 18 possible stimulus conditions from all possible combinations of three target eccentricities ( $e=1^{\circ }$ , $3^{\circ }$ , and $9^{\circ }$ ) and six target-mask situations that included five SOAs ( SOA=−50 , 0 , 50 , 92 , or 142 ms) and one situation when no mask was present. SOA was defined as the mask’s onset time relative to the target’s onset time, and for convenience, the no-mask situation is sometimes denoted by an SOA= ∞ . In each trial, the subjects’ task was to take their time to report, after the offset of the last stimulus component in a trial, whether the target diamond is missing its left or right corner by pressing a left or right button, respectively. An eye tracker monitored observers’ fixation locations. As explained by Figure 2, trials of different conditions were randomly interleaved. With 40 trials for each of the 18 stimulus conditions, each observer performed 720 testing trials in eight blocks of 90 trials each, with breaks between the blocks.

### Experimental Equipment

The visual stimuli were displayed using a VIEWPixx/EGG display screen from VPixx Technology at 120 Hz frame rate. The eye tracker was CRS LiveTrack Lightning which sampled at 50 Hz. The experiment was conducted in a dimly lit small room, with the white background (with luminance 100 $cd/m^{2}$ ) of the display screen as the main source of illumination.

### Procedure

Each observer sat in front of the display with a viewing distance of 64 cm maintained by a chin stand. At the beginning of each experimental session, a fixation cross appeared near the center of the display and stayed on the display throughout the session. At the start of each trial, two horizontal bars appeared below the fixation cross (see Figures 1 and 2). They had the same displacement vertically from the fixation cross. Their center of mass was below the fixation cross to coincide with the center location of the upcoming target and mask at eccentricity $e=1^{\circ }$ , $3^{\circ }$ , or $9^{\circ }$ . They thus served to inform the observer of the upcoming target location. At 300 ms afterwards, the text “Look here” (see Figures 1 and 2) appeared next to the fixation cross to remind observers of the fixation requirement that gaze must be directed to the fixation cross for the whole duration of stimulus presentation in each trial. To proceed with the trial, the observer pressed a button, triggering the disappearance of “Look here. Starting from 600 ms after this button press, the first stimulus component—target, mask, or both, depending on the SOA—would appear as soon as the observer’s fixation satisfied a fixation criterion verified by the eye tracker. This criterion required that the gaze position was within 1.5 degree horizontally and vertically from the center of the fixation cross continuously for the preceding 200 ms. If this criterion was not satisfied within 1500 ms from the button press, the trial would proceed forward with the onset of the first stimulus component, although the trial was later regarded as invalid and excluded from the data analysis. This first stimulus component disappeared after 25 ms. For trials with a mask and a nonzero SOA=−50,50,92,or142ms , the second stimulus component—target or mask depending on whether SOA is negative—appeared at |SOA|−25 ms after the offset of the first stimulus component and disappeared 25 ms afterwards. No second stimulus component followed the first one if the trial was a zero SOA trial or a no-mask trial. After the offset of the last stimulus component in a trial (see Figure 2), the observer could take his/her time to press a left or right button to report whether the left or right corner of the target diamond was missing. This button press triggered the start of the next trial with the onset of the horizontal bars to indicate to the observer the location of the target in the next trial.

### Sizes of Various Stimulus Components

In the image containing the target and the mask for $e=1^{\circ }$ , the sizes of various spatial components are the same as, or similar to, those in Enns and Di Lollo (1997) whose stimuli were viewed foveally. These sizes were scaled up for $e=3^{\circ }$ and $e=9^{\circ }$ by scale factors 2.5 and 7, respectively, to compensate for visual crowding for peripheral stimuli. These scale factors were determined during pilot experiments such that, for typical observers, task performance accuracies for different eccentricities were similar for each of these two SOA values, SOA=0 and SOA=300 ms, for which the masking effect was absent or weak by foveal viewing in Enns and Di Lollo (1997).

There was a large variability across subjects in their task performance accuracies, such that the standard deviation of the accuracies, which are by definition (see later) within [0,1] , often reached ∼0.15 for a given condition. To ensure that our scaling factors are optimal, the pilot experiments to determine these factors involved 47 pilot subjects, including the authors, from the same general population of subjects in our university area. However, the large variability among subjects meant that our scale factors could not be sufficiently adequate for atypical subjects, defined as those who had statistically nonequivalent (by unpaired permutation test using valid trials) performance accuracies between any two out of the three eccentricities for the SOA = 0 or for the no mask condition (this occurred in nine or three out of the 30 subjects in the zero SOA or no mask condition, respectively). To compensate for this problem, whenever relevant, the masking effects were examined also by normalizing each accuracy by that of the same subject in the zero SOA or the no mask condition (see details later). Removing the atypical subjects from the data analysis gave qualitatively the same conclusions in this paper.

Care was taken so that the scale factors were not too large to obscure any possible masking effects (at larger eccentricities) for positive SOA values less than SOA=300 ms (see discussions later).

In detail, the sizes of the various components of the target, mask, and contextual elements are completely specified by the quantities named in Figure 1, and listed in Table 1.

**Table 1.** Spatial extent of the stimuli in degrees (for $f_{w}$ , $f_{l}$ , l , k , e , g ) or in multiples of g (for c , d , m , h , $b_{l}$ , $b_{w}$ ).

$f_{w}$ : width of the line in the fixation cross	$0.05^{\circ }$		
$f_{l}$ : length of the line in the fixation cross	$0.29^{\circ }$		
l : height of the text “Look here”	$0.73^{\circ }$		
k : horizontal extent of the text “Look” and “here”	$\approx 2^{\circ }$		
e : eccentricity	$1^{\circ }$	$3^{\circ }$	$9^{\circ }$
g : gap between the target and mask	$0.02^{\circ }$	$0.05^{\circ }$	$0.14^{\circ }$
c/g ( c : vertical extent of the missing corner in the target)	8.5		
d/g ( d : vertical extent of the target diamond)	31		
m/g ( m : thickness of the masking frame)	10		
h/g ( h : shortest distance between the horizontal bar and the target’s center)	100		
$b_{l}/g$ ( $b_{l}$ : length of the horizontal bars)	18		
$b_{w}/g$ ( $b_{w}$ : width of the horizontal bars)	1.7		

### Data Analysis

To obtain our results, we exclude all trials in which the subjects did not fixate properly (see Materials and method). These excluded trials are called invalid trials, and the other trials are valid trials. Among our n=30 observers, the minimum and average fractions of trials that were valid were 0.918 and 0.99 , respectively.

We define accuracy $A_{s,e,SOA}$ as the fraction of the valid trials that subject s performed correctly for trials at eccentricity e and at a particular SOA value (SOA =∞ denotes the no-mask condition). Sometimes, to examine the effect of SOA, we also define the normalized accuracy as $A_{s,e,SOA}$ divided by the corresponding accuracy by the same observer s and at the same eccentricity e at zero SOA or with no mask, as will be specified in the results. Averaged across observers, accuracies (normalized or otherwise) between two conditions, one with $\left(e,\mathrm{SOA})=(e_{1},{\mathrm{SOA}}_{1}\right)$ and the other with $\left(e,\mathrm{SOA})=(e_{2},{\mathrm{SOA}}_{2}\right)$ , are said as significantly different from each other if the probability p value gives p<.05 by a matched-sample permutation test between the respective lists of the accuracies across the subjects for the null hypothesis that the two lists of accuracies are statistically equal. Qualitative conclusions in this paper are unchanged if *t*-tests were used instead.

Gender difference has been found in backward masked vernier tasks (Shaqiri et al., 2018) using data from hundreds of subjects. Perhaps partly because we used fewer subjects, we found no significant gender difference in any of our masked conditions after corrections for multiple comparisons. However, in our no mask condition at $e=1^{\circ }$ , males had a lower mean accuracy of 0.945 than the mean accuracy of 0.986 by females, with a p -value of p=.025 . Given our focus on masked conditions, we report our results below using all subjects’ data regardless of gender.

## Results

### The Strongest Masking was Backward Masking at 50 ms SOA at All Three Eccentricities

**Figure 3.**
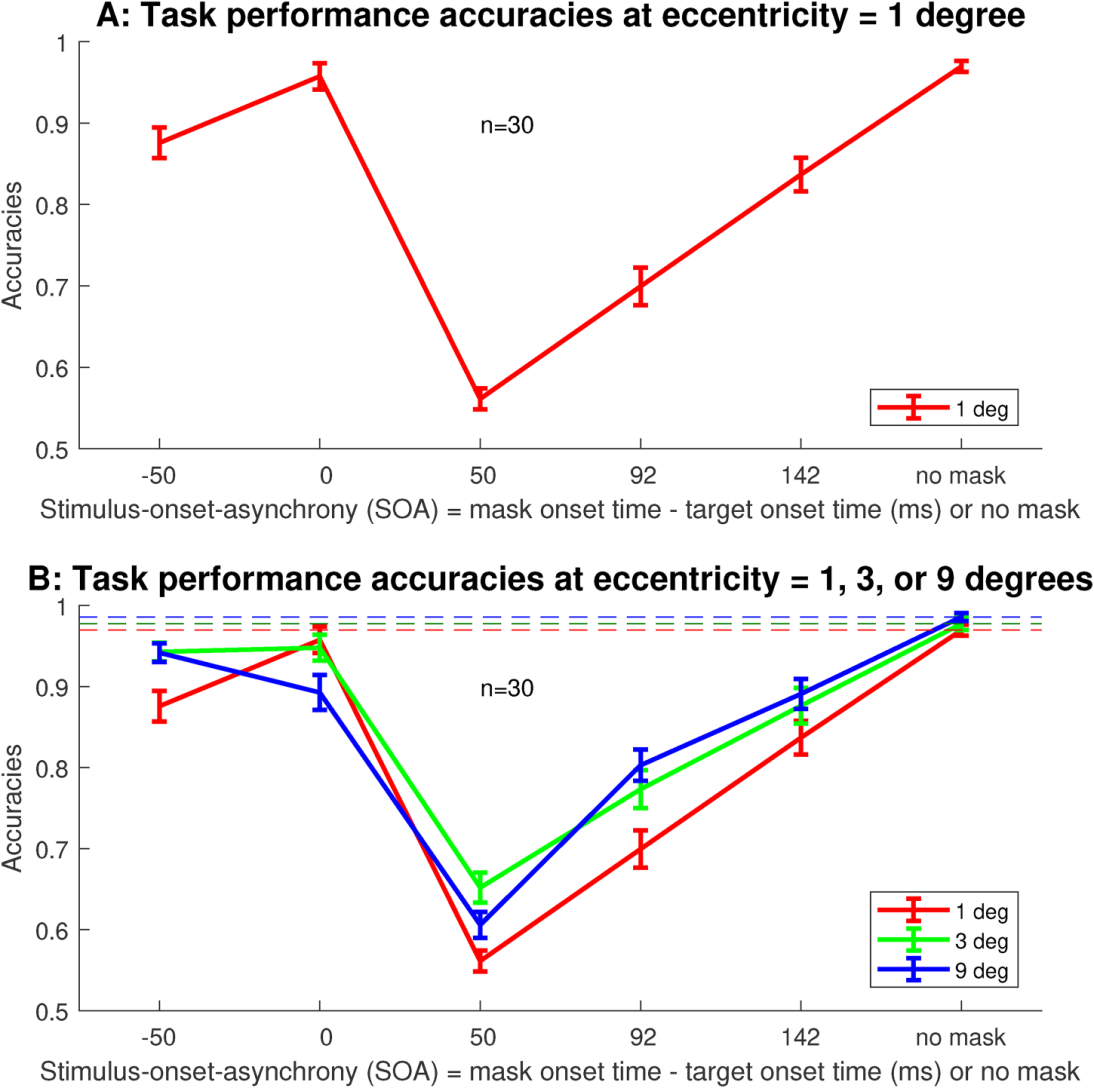
Performance accuracies for target discrimination averaged across n=30 observers. A: the accuracies at eccentricity $e=1^{\circ }$ . B: the accuracies for all three eccentricities $e=1^{\circ }$ , $3^{\circ }$ , and $9^{\circ }$ . Error bars are standard errors of the mean across observers. Red, green, and blue horizontal dashed lines mark the mean accuracies when there was no mask for eccentricities $e=1^{\circ }$ , $3^{\circ }$ , and $9^{\circ }$ , respectively.

Averaged across observers, the masking behavior at our smallest eccentricity $1^{\circ }$ was qualitatively very similar to that observed in the previous study by Enns and Di Lollo (1997) with foveal viewing of the stimulus, see Figure 3A. In particular, the average task accuracy was nearly perfect at SOA=0 ms, and was statistically not different ( p=.23 ) from that without the mask. However, task accuracy sank to near the chance level of 50% at SOA=50 ms. As SOA increases from 50 to 92 and 142 ms, the masking effect weakened. Compared to backward masking at SOA =50 ms, forward masking at SOA=−50 ms was nearly negligible, with task accuracy nearly 90%. This accuracy as a function of SOA followed the U-shaped curve that is characteristic of metacontrast masking with foveal viewing (Kahneman, 1968; Enns and Di Lollo, 1997; Breitmeyer and Öğmen, 2006).

**Figure 4.**
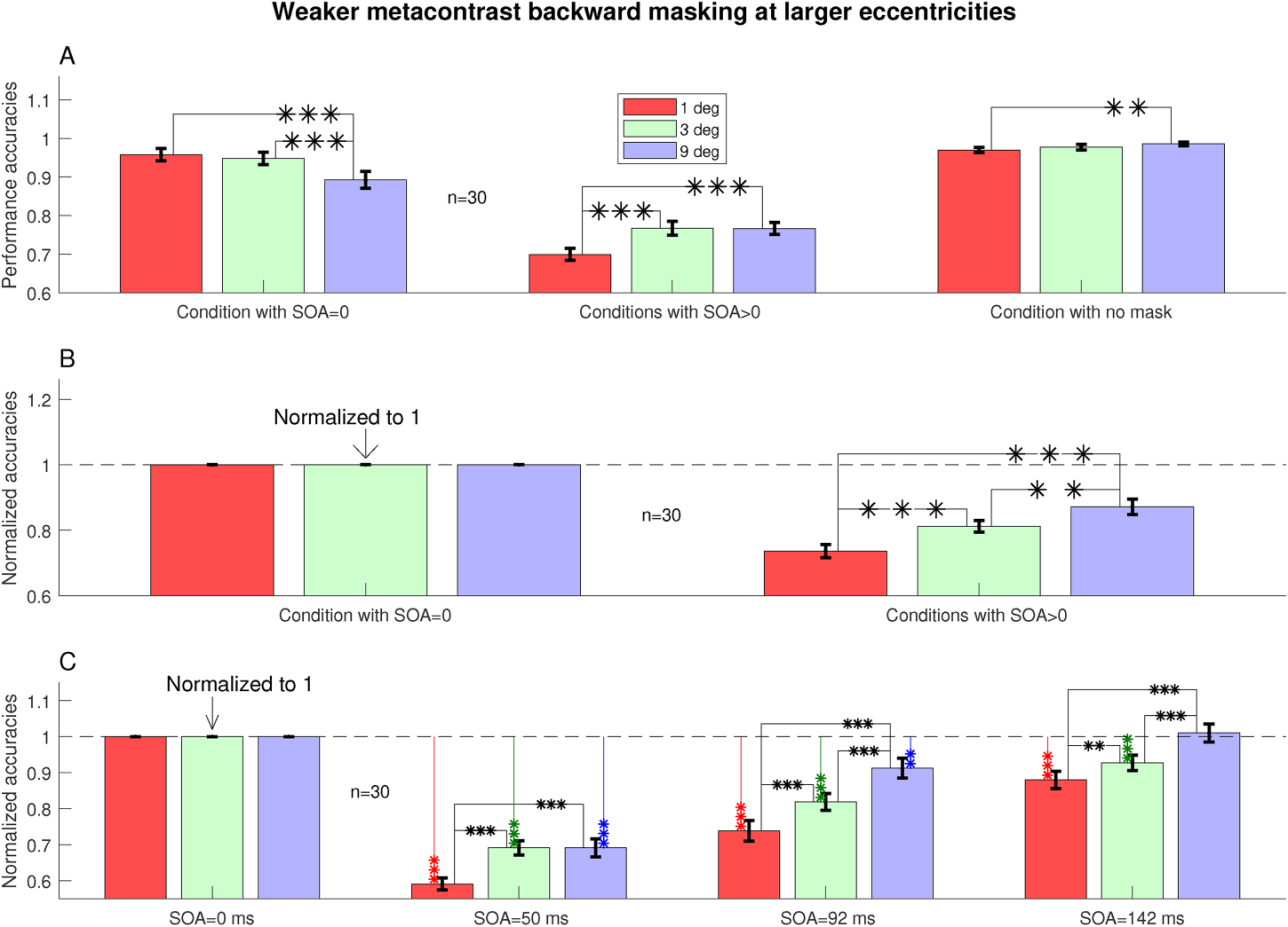
Backward masking (SOA >0 ) relative to simultaneous masking (SOA =0 ) shows that backward masking was weaker at larger eccentricities. A: observer averaged accuracies in conditions SOA =0 , no mask, and SOA >0 (obtained by averaging the accuracies across the three positive SOAs, 50, 92, and 142 ms, for each observer before taking the average across observers). Error bars denote the standard error of the mean (across observers). Two data bars linked by lines with one, two, or three ‘*’ indicate that the two corresponding accuracies are significantly different from each other by a matched-sample permutation test with .01≤p<.05 , .001≤p<.01 , and p<.001 , respectively. B replots the results for the masked conditions in A using normalized accuracies (obtained by dividing each accuracy of an observer by his/her accuracy at the same eccentricity at SOA =0 ). C is B replotted after detailing each positive SOA. A data bar linked by one, two, or three ’*’s to the dashed horizontal line indicates that the normalized accuracy is significantly different from the corresponding (normalized) accuracy for zero SOA by a matched-sample permutation test with 0.01≤p<.05 , .001≤p<.01 , or p<.001 , respectively.

Figure 3B plots the task accuracies at all the three eccentricities, $1^{\circ }$ , $3^{\circ }$ , and $9^{\circ }$ . Qualitatively, accuracies as a function of SOA followed a similar U-shaped curve for each eccentricity. For each e , the accuracy was lowest at SOA=50 ms, and was near perfect for zero SOA, negative SOA, and when there was no mask. Hence, metacontrast masking was present at all three eccentricities. Note that we have aimed to enlarge the stimulus for the larger eccentricities to compensate for all or most of the crowding effect. In the no mask condition, this enlargement made the task accuracies (averaged across subjects) 97% , 98% , and 98.6% for $e=1^{\circ }$ , $3^{\circ }$ , and $9^{\circ }$ , respectively, with the accuracy at $e=9^{\circ }$ very slightly but significantly larger than that at $e=1^{\circ }$ ( p=.008 , see also Figure 4A). In the zero SOA condition, this same enlargement made the corresponding accuracies 96% , 95% , and 89.6% , with the accuracy at $e=9^{\circ }$ significantly lower than those at lower eccentricities ( p≤0.0005 , Figure 4A). This suggests that it is difficult to find a single stimulus enlargement scaling to compensate for visual crowding in both the zero SOA and no-mask conditions (so that the mean accuracy at each $e>1^{\circ }$ was statistically equal to that at $e=1^{\circ }$ in both the zero SOA and no-mask conditions). Particularly for $e=9^{\circ }$ , our stimulus size scaling was such that the compensation for crowding for zero SOA was largely but not 100% complete.

### Backward Masking was Weaker at Larger Eccentricities

Figure 4 examines more closely the differences between different eccentricities for backward masking with SOA >0 . Pooling data at all the three positive SOAs (at 50, 92, and 142 ms) together, Figure 4A shows that the task performance for $3^{\circ }$ and $9^{\circ }$ eccentricities were better ( p<.0001 ) than that at $1^{\circ }$ eccentricity at SOA >0 . Hence, the backward masking was weaker at larger eccentricities. This weaker masking at larger e was not an artifact from too much scaling up of the stimulus at higher eccentricities to compensate for visual crowding, at least for $e=9^{\circ }$ . This is because, compared to the accuracy at $e=1^{\circ }$ , the accuracy at $e=9^{\circ }$ was larger ( p<.0001 ) at SOA >0 even though it was smaller ( p=.0002 ) at SOA =0 .

To examine the backward masking (at SOA >0 ) relative to simultaneous masking (at zero SOA) more closely, we define normalized accuracy by each observer s at eccentricity e and SOA as $A_{s,e,SOA}/A_{s,e,SOA=0}$ , by dividing each accuracy $A_{s,e,SOA}$ at any SOA by its counterpart $A_{s,e,SOA=0}$ at zero SOA. This normalized accuracy at SOA >0 significantly increased with every eccentricity increase, from $e=1^{\circ }$ to $e=3^{\circ }$ ( p<.0001 ) and from $e=3^{\circ }$ to $e=9^{\circ }$ ( p=.002 , Figure (4)B). Hence, the backward masking was strongest at the smallest eccentricity $e=1^{\circ }$ and weakest at the largest eccentricity $e=9^{\circ }$ .

Examining the three SOAs (50, 92, and 142 ms) individually, Figure 4C shows that the masking was strongest at $e=1^{\circ }$ ( p≤.0083 ) at all the three SOAs, while masking was weaker at $9^{\circ }$ than at $3^{\circ }$ at the two larger SOAs ( p≤.0002 ). Furthermore, by the largest SOA of 142 ms, the accuracy at the largest eccentricity $e=9^{\circ }$ recovered to be statistically not different from the corresponding accuracy at zero SOA ( p=.35 ). Meanwhile, this complete recovery by SOA of 142 ms did not occur at smaller eccentricities $e<9^{\circ }$ ( p<.0005 ). Hence, mechanisms to make backward masking stronger than simultaneous (zero SOA) masking decayed with the increasing SOA of the mask, and this decay was faster at the largest eccentricity $e=9^{\circ }$ .

### Forward masking is also weaker at larger eccentricities

**Figure 5.**
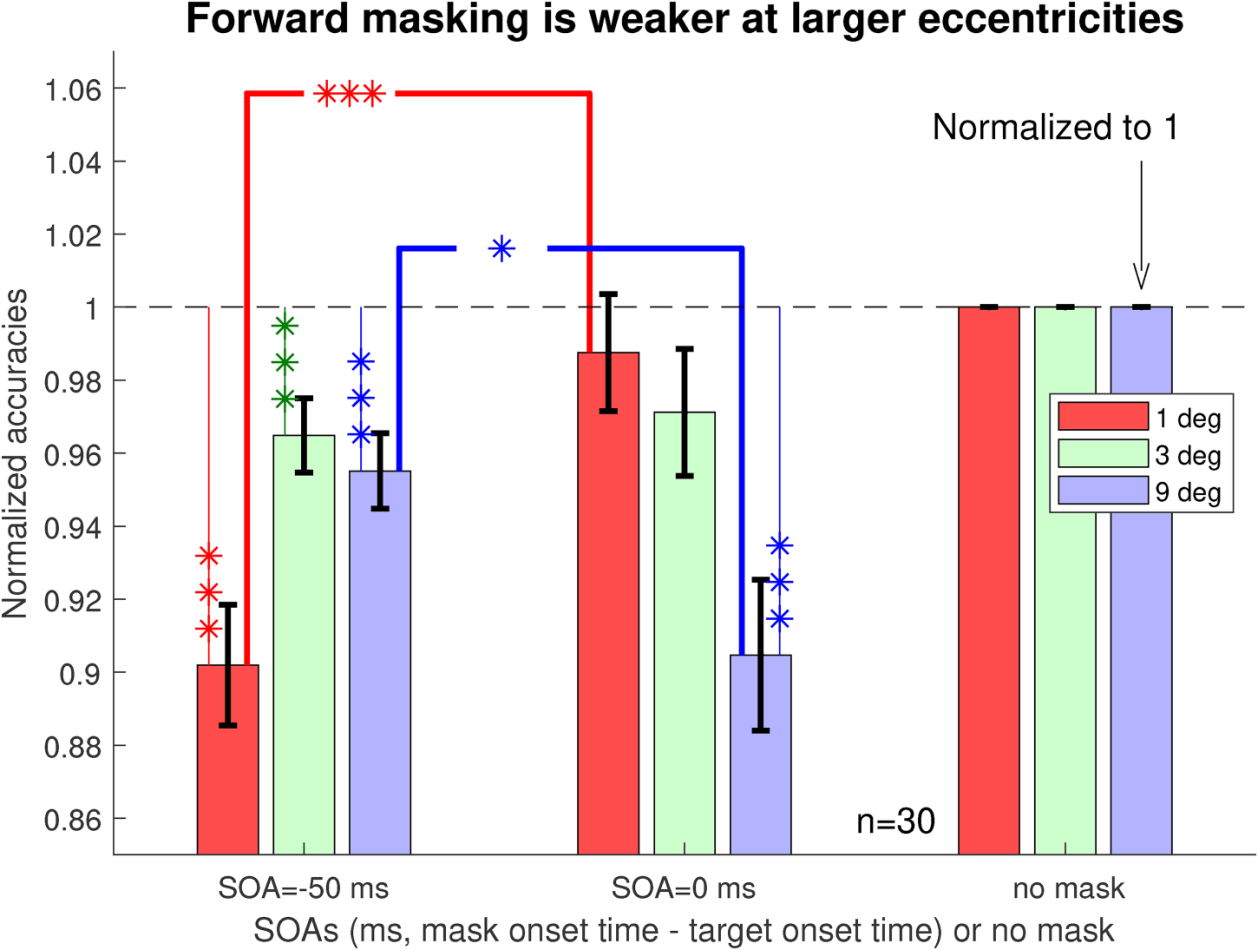
Forward masking is weaker at larger eccentricities. Shown are task accuracies at zero and negative SOAs relative to those in the no-mask conditions, using normalization so that each original accuracy $A_{s,e,SOA}$ becomes the normalized accuracy with a value $A_{s,e,SOA}/A_{s,e,\mathrm{no}\text{-}\mathrm{mask}}$ before averaging over n=30 observers. A data bar linked by one, two, or three ’*’s to the dashed horizontal line indicates that the normalized accuracy is significantly different from the corresponding (normalized) accuracy in the no-mask condition by a matched-sample permutation test with .01≤p<.05 , .001≤p<.01 , or p<.001 , respectively. Two data bars linked by one, two, or three ’*’s indicate that the two corresponding accuracies are significantly different from each other by a matched-sample permutation test with .01≤p<.05 , .001≤p<.01 , or p<.001 , respectively.

Forward masking is much weaker than backward masking at all eccentricities (see Figure 3). Meanwhile, a closer examination by Figure 5 shows that there was a dependence on eccentricity when we compare forward masking (SOA <0 ) with simultaneous masking (SOA =0 ). By this comparison, the (normalized) accuracy at SOA =−50 ms was worse ( p=.0001 ), about the same ( p=.38 ), or better ( p=.013 ) at eccentricity $e=1^{\circ }$ , $e=3^{\circ }$ , or $e=9^{\circ }$ , respectively. In particular, for the smallest $e=1^{\circ }$ , there was no simultaneous masking effect (since the (normalized) accuracy at zero SOA was not statistically different ( p=.23 ) from that with no mask) but there was a significant forward masking effect ( p<.0001 ). In contrast, for the largest $e=9^{\circ }$ , there was a significant simultaneous masking or crowding effect at zero SOA ( p<.0001 ). However, when the mask appeared before the target at SOA =−50 ms, although the masking effect remained very significant ( p<.0001 ), it is relatively weaker ( p=.013 ) when compared with simultaneous masking at zero SOA. We will discuss this more in the next section.

## Summary and discussion

Metacontrast masking is manifested by a U-shaped curve of target recognition performance as a function of SOA, this curve dips substantially around SOA=50−100 ms and saturates for SOA too small (zero or negative) or too large. Assessing metacontrast masking by target recognition performance at positive SOAs relative to the performance at zero SOA, we found that metacontrast masking is present at all the three different eccentricities $e=1^{\circ }$ , $3^{\circ }$ , and $9^{\circ }$ and that this masking is progressively weaker at larger eccentricities. Weaker metacontrast masking for larger eccentricities is predicted by the CPD if the masking works by interfering with the top-down feedback to aid visual discrimination, since the CPD states that such feedback is weaker in the peripheral visual field so that visual recognition should rely less on such feedback.

Our conclusion that metacontrast is weaker at larger eccentricities was reached after we took care of the effects of visual crowding and visual attention. We compensated for crowding by enlarging the visual inputs for larger eccentricities so that target discrimination accuracy was comparably near 100% at SOA =0 (and SOA =300 ms during our pilot experiments) across the three eccentricities. Since an overcompensation could cause weaker masking as an artifact, we note that our compensation for particularly the largest $e=9^{\circ }$ was a (slight) under- rather than overcompensation. In particular, target discrimination at zero SOA was slightly but significantly worse at $e=9^{\circ }$ than those at the two smaller e ’s (see Figures 3B and 4A). Is there any overcompensation at our intermediate $e=3^{\circ }$ to affect our conclusion? At zero SOA, $e=3^{\circ }$ and $e=1^{\circ }$ had statistically equivalent ( p=.12 ) accuracies of 95% and 96% , respectively (Figure 4A). Hence, at SOA >0 , the weaker masking at $e=3^{\circ }$ than $e=1^{\circ }$ ( p<.0001 ) was unlikely caused by an overcompensation, whereas the stronger masking at $e=3^{\circ }$ than $e=9^{\circ }$ ( p=.002 , Figure4B) argues against an overcompensation at $e=3^{\circ }$ . The effects of visual attention in masking are caused by uncertainty about the location of an upcoming target, as such uncertainty makes attention not properly focused on the target when it appears and impairs target recognition for metacontrast masking as well as OSM (Enns and Di Lollo, 1997). We controlled for this by cueing the target location before the stimulus onset in each trial using the horizontal bars.

It is a long-standing idea that the brain uses both feedforward and feedback processes for object recognition (MacKay, 1956; Carpenter and Grossberg, 1987; Li, 1990; Kawato et al., 1993; Dayan et al., 1995; Yuille and Kersten, 2006). The feedback component is expected to feature more heavily, and multiple iterations of feedforward and feedback processes are often needed, in more challenging situations such as brief, noisy, partially occluded, and/or ambiguous sensory inputs. OSM (Enns and Di Lollo, 1997; Di Lollo et al., 2000) is a very illustrative manifestation of such interactions between feedforward and feedback signals. The CPD additionally proposes that the feedback component is weaker or absent in the peripheral visual field (Zhaoping, 2017, 2019), since computational resources in the brain are limited. Although this study uses the CPD to investigate whether metacontrast masking interferes with the feedback processes, since the CPD is still a recent hypothesis that is yet to be further tested, this study can also be seen as using metacontrast masking to test the CPD if this masking is assumed to involve interference of the feedback component. Our findings indicate that the CPD and the idea of feedback interference by metacontrast masking are consistent with each other.

Since its recent proposal (Zhaoping, 2017), support for the CPD has come from experimental confirmations of its predicted visual illusions in the peripheral visual field (Zhaoping and Ackermann, 2018; Zhaoping, 2020). These predictions arise because a lack of sufficient feedback process to aid visual recognition in ambiguous situations makes peripheral vision vulnerable to misleading visual inputs, in light of the information bottleneck starting from V1’s output so that perceptual decisions in higher brain areas are made from scanty information sent from V1. Stronger feedback in central vision to aid recognition is supported by a stronger bias to perceive, among multiple plausible perceptual outcomes (in situations of ambiguous perception), the outcome that is more consistent with expectations by brain’s internal models of the visual world (Zhaoping, 2017). The interaction between the feedforward and feedback process can be paraphrased as Feedforward-Feedback-Verify-reWeight (FFVW) (Zhaoping, 2017, 2019) as follows: initial sensory inputs feedforward to initiate candidate hypotheses about the visual scene; higher brain areas synthesize from the brain’s internal models would-be visual inputs consistent with each hypothesis; these would-be visual inputs are fed back to V1 (which has been hypothesized (Zhaoping, 2019) as before the start of the information bottleneck along the visual pathway) to compare with the actual visual inputs; and the weight of each hypothesis for becoming the perceptual outcome is increased or decreased if the match between the would-be and actual inputs is relatively better or worse, respectively. This FFVW process should veto perceptual hypotheses that are suggested by V1’s responses to retinal inputs but are inconsistent with the brain’s internal models. Accordingly, reversed depth from contrast-reversed random-dot stereograms or flip tilt illusions are typically not perceived in central vision (but visible in peripheral vision) (Zhaoping and Ackermann, 2018; Zhaoping, 2020). The target made invisible by metacontrast masking by SOA >0 is also presumably vetoed due to a conflict between the would-be input containing the target signals and the actual input arising from the trailing mask, especially when the stimuli are hard to be interpreted as arising from an apparent motion or updating from the target’s shape or position to the mask’s shape or position (Kahneman, 1968; Goodhew et al., 2013). Indeed, many subjects, including the authors, reported that the target diamond was often invisible, or appeared as a complete diamond (without any corner missing, perhaps because the mask’s contour was seen as the target’s contour). The perceptual impression was qualitatively different from that of seeing too many contour fragments crowded together like in visual crowding. In depth perception of random-dot stereograms in central vision, it has been demonstrated that stimulus components (from dichoptically contrast-reversed dots) that are normally vetoed (and thus invisible) can nevertheless enhance or sometimes degrade another perceptual outcome arising from other stimulus components (from dichoptically contrast-matched dots) (Zhaoping, 2021). This manifests a complex interaction between the feedforward and feedback processes.

Sensitivity to the physical distance between target contours and mask contours (Breitmeyer and Öğmen, 2006; Enns and Di Lollo, 1997) and inhibition of V1 responses to the target by a spatiotemporally nearby mask (Macknik and Livingstone, 1998; Macknik and Martinez-Conde, 2007) have provided perhaps the strongest support to the idea that metacontrast masking interfered mainly with feedforward mechanisms for target recognition. However, for backward masking, although V1 neural responses to the target are most inhibited by masks when the interstimulus interval (ISI) between the target’s offset and the mask’s onset is zero (Macknik and Livingstone, 1998; Macknik and Martinez-Conde, 2007), strongest perceptual masking typically occurs for an ISI >0 for brief targets (Kahneman, 1968; Enns and Di Lollo, 1997; Macknik and Martinez-Conde, 2007). Although V1’s responses to the target is more inhibited by a preceding rather than a succeeding mask (Macknik and Livingstone, 1998), backward masking is much stronger than forward masking perceptually. These observations add to the observations that early neural responses to a brief target are often little affected by succeeding masks with an ISI >50 ms (Bridgeman, 1980; Jeffreys and Musselwhite, 1986; von der Heydt, 2022) to suggest that metacontrast masking mainly interferes with the feedback processes for target recognition. The fact that the peak masking effect occurs at SOA around 40–100 ms, known since decades ago from similar and related masking effects (Kahneman, 1968; Westheimer et al., 1976; Ng and Westheimer, 2002; Di Lollo et al., 2004; Breitmeyer and Öğmen, 2006), is in line with a 30–40 ms latency between the feedforward and feedback components in visual cortical areas of monkeys suggested by neurophysiological data (Chen et al., 2014, 2017; Yan et al., 2018; Klink et al., 2017).

Our forward masking, when SOA is −50 ms, is much weaker than backward masking. Such masking most likely affects mainly the feedforward mechanisms for target recognition, such as the inhibition of V1 responses to the target’s onset by V1 responses to the mask’s offset (Macknik and Livingstone, 1998; Macknik and Martinez-Conde, 2007). However, compared with simultaneous masking (at zero SOA) at the same eccentricity (Figure 5), forward masking is stronger at $e=1^{\circ }$ but weaker at $e=9^{\circ }$ . This contrast between $e=1^{\circ }$ and $e=9^{\circ }$ may be understood by including additionally the CPD that peripheral and central vision are mainly for looking and seeing, respectively (Zhaoping, 2019). Looking is to attentionally select a visual location for deeper processing by shifting our gaze or attentional spotlight to it. This selection can be guided by both endogenous and exogenous factors. Our endogenous guidance is via the cueing by the horizontal bars to inform observers about the location of the upcoming target. In addition, an exogenous guidance can come from the salient onset of the mask, and, as demonstrated previously (Nakayama and Mackeben, 1989), such an exogenous flash at the expected location of the upcoming target can additionally boost target discrimination performance as an attentional cueing effect. Due to the CPD, exogenous saliency effects are expected to be stronger for more peripheral visual locations (Zhaoping, 2014, 2019). This explains a weaker forward masking at larger eccentricities observed in our data.

In summary, according to our data, the CPD, which hypothesizes that top-down feedback for object recognition is weaker in the peripheral visual field, and the idea that metacontrast (backward) masking mainly interferes with feedback mechanisms for object recognition are mutually supportive of each other. This could be tested further in future studies using other stimulus and task examples of metacontrast masking.

This study is also another demonstration showing that peripheral vision cannot be equated with central vision once the visual input size is scaled up to compensate for a reduction in the cortical magnification factor (the extent of the retinotopic V1 receiving inputs from one unit of solid visual angle) (Rovamo and Virsu, 1979; Koenderink et al., 1978). One can apply the CPD to other visual phenomena to infer the underlying neural mechanisms (Zhaoping, 2019). For example, visual hyperacuity (Westheimer, 1981) is the human visual ability to resolve spatial details finer than the image sampling resolution on the retina. This hyperacuity (for a 500 millisecond viewing duration) deteriorates from fovea to periphery faster than suggested by V1’s cortical magnification factor (Westheimer, 1982; Fendick and Westheimer, 1983). This faster deterioration suggests, according to the CPD, that top-down feedback is likely involved to achieve this hyperacuity feat. Indeed, at fovea, this acuity worsens with shorter viewing durations (Westheimer and McKee, 1977), presumably because a shorter viewing hinders or prevents the feedback process to function (as suggested by an example of depth perception at fovea (Zhaoping, 2021)), and, if so, the CPD predicts that, at a more peripheral location, hyperacuity should suffer less from a shorter viewing duration. Many other visual discrimination tasks, on which human performance deteriorates with visual field eccentricity faster than suggested by a reduced V1 cortical magnification factor (Strasburger et al., 2011), could be examined analogously in this light.
